# Optical Coherence Tomography Angiography to Better understand Glaucoma

**DOI:** 10.5005/jp-journals-10028-1219

**Published:** 2017-08-05

**Authors:** Gábor Holló

**Affiliations:** Head, Glaucoma and Perimetry Unit, Department of Ophthalmology Semmelweis University, Budapest, Hungary

**Keywords:** Glaucoma, Optical coherence tomography angi-ography, Optic nerve head, Perfusion, Peripapillary retina.

## Abstract

The term optical coherence tomography angiography (OCTA) comprises different OCT-based technologies which all allow noninvasive assessment of retinal perfusion, based on moving red blood cells. The main areas where OCTA is currently used are investigation of perfusion and vascular structure of the macular retina (e.g., in macular degenerations and diabetic macular diseases) and the disk and peripapillary retina (in glaucoma and other optic disk diseases). The current editorial provides a brief overview on the potential of OCTA and its use to measure perfusion in the peripapillary retina in glaucoma.

**How to cite this article:**

Holló G. Optical Coherence Tomography Angiography to Better understand Glaucoma. J Curr Glaucoma Pract 2017;11(2):35-37.

## WHAT IS OPTICAL COHERENCE TOMOGRAPHY ANGIOGRAPHY?

The term optical coherence tomography angiography (OCTA) comprises different OCT-based methods, which all offer noninvasive and cross-sectional assessment of the perfusion in the retina or other eye tissues (e.g., cornea and filtering bleb). Various OCTA instruments manufactured by several manufacturers are currently used in clinical practice. All systems offer perfusion images, however, currently not all instrument and software versions provide numerical measurements. Due to the technical differences, no conversion of the measured results between the different systems is possible. Most commercially available OCTA systems are based on split-spectrum amplitude-decorrelation algorithm.^1^ This algorithm detects red blood cell movement independently from the direction of the movement. Thus, in contrast to fluorescein angiography, information in OCTA arrives from the red blood cells and not the plasma; this may result in differences in clinical interpretation, mainly when the macula is investigated. It is important to note that lack of perfusion with OCTA does not necessarily mean missing or obstructed vessels, or lack of capillary perfusion; in contrast, it simply means that at the time of image acquisition, no moving red blood cell was present in the location of the low perfusion or nonperfusion OCTA areas.

**Fig. 1:**
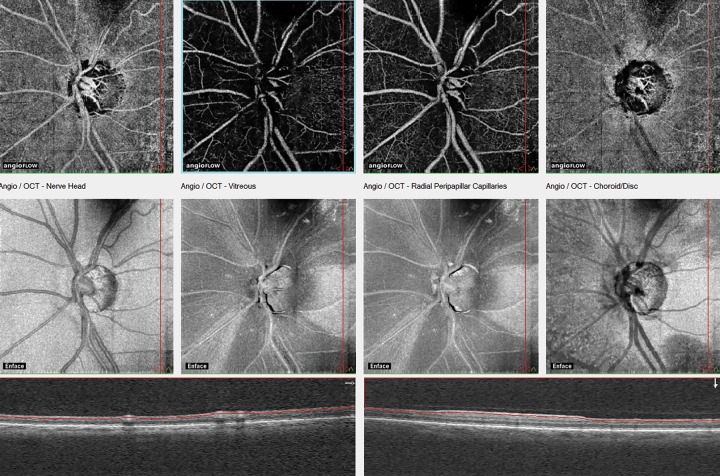
*En face* optical coherence angiography images and the corresponding en-face OCT images of four layers of the disk and peripapillary retina in severe glaucoma. The measuring ellipse for numerical perfusion measurement is not fitted on the image. The layers from left to right reflect the vitreous level, the superficial and deep sections of the radial peripapillary capillaries layers, and the superficial choroid. In the radial peripapillary capillaries layer, the structural damage (wide retinal nerve fiber layer dropouts) spatially correspond with the reduction of perfusion

For glaucoma research and glaucoma practice, the most informative parameters are peripapillary angioflow density (the perfusion area expressed in the percent of the total examined peripapillary area and its predefined sectors, respectively) and whole image angioflow density (the perfusion area expressed in the percent of the total examined image area).^2^ Though OCTA of the disk area is technically available in all systems, the presence of the large vessels, and the variability and complexity of the three-dimensional structure of the disk makes it difficult to interpret the measurement results. In OCTA, both the perfusion and the corresponding structural information *(en face* OCT) of the different retinal layers are imaged and measured respectively, on the same image (Fig. 1). This allows the combined analysis of the images and the measurements, and helps the investigator finding correspondence between a structural abnormality and its perfusion.^3,4^ For diseases involving the retinal nerve fiber layer (e.g., glaucoma), OCTA of the radial peripapillary capillaries layer provides the most useful information. This layer is identical to the retinal nerve fiber layer on the structural *(en face)* OCT image.

## OPTICAL COHERENCE TOMOGRAPHY ANGIOGRAPHY IN GLAUCOMA MANAGEMENT

It has been shown that the diagnostic accuracy of peri-papillary and whole image angioflow density for separation of glaucoma eyes from normal eyes is similar to that of the retinal nerve fiber layer thickness.^1^ In the radial peripapillary capillaries layer, peripapillary angioflow density shows strong correlation with the retinal nerve fiber layer thickness.^3^ The measurement reproducibility is favorable and independent from the severity of glaucoma. This means that measurements made in advanced glaucoma are as reliable as those made on healthy and early glaucoma eyes.^3^ The perfusion of the peripapillary OCTA sectors shows strong relationship with the sensitivity and defect values of the spatially corresponding visual field areas.^5^ Recently, in a proof-of-concept study, it was shown in untreated high pressure open-angle glaucoma and ocular hypertensive eyes that at least 50% intraocular pressure (IOP) reduction to IOP of less than 18 mm Hg causes a significant increase of the peripapillary angioflow density.^6^ This means that there is a relationship between IOP and peripapillary perfusion. However, the detailed evaluation of the relationship and its potential applicability for glaucoma care remains to be specified.

## FUTURE OF OCTA IN GLAUCOMA CARE

The OCTA entered clinical glaucoma practice 3 years ago. Thus, the time elapsed since its introduction was too short compared to the time necessary for the development of glaucomatous progression. Therefore, currently no information is available on the long-term changes of peripapillary angioflow density in treated glaucoma. It is also unknown whether OCTA can provide better or additional information on progression of glaucoma compared to the currently established structural OCT parameters (the retinal nerve fiber layer thickness and the macular inner retinal thickness). This limitation, however, is temporary. In the following years, we will see if OCTA can be usefully applied to detect glaucomatous progression earlier, to measure glaucomatous progression more reliably, or to find markers which differentiate eyes with optimal and suboptimal pressure control. Since OCTA is one of the most exciting and promising new technologies in glaucoma, it desires attention from both glaucoma specialists and general ophthalmologists treating glaucoma patients.
